# Notices

**Published:** 1863-12

**Authors:** 


Personal Notice.—The editor has opened an office for consultation and business temporarily, at 831 Broadway, New-York, where he may be found daily, from ten to one o'clock p.m. He does not wish to be seen at any other time, for the present. Persons who can not call upon him within ten hours specified, are requested to address a note to him a day or two beforehand, to arrange an interview for some other hour. By this arrangement, our business will be concentrated to a smaller portion of the day, leaving us more time to write for the Journal without innumerable interruptions; to play with the children, and look in at the shop-windows incog, in glorious old Broadway. It is not good for any body's health to be confined to one room from daylight to bedtime ready for calls, as has been the case with us for so many years past in this city; and although we can not remember the day when we omitted to eat a meal for want of an appetite, and are generally as lively as a cricket and playful as a little lamb or kitten, still, by the new arrangement, we are satisfied that our readers, our patients, and our children will be benefited; and our health and happiness be promoted; and all this, without incommoding any one in any way.

"The Soul of Things." By Wm. and Elizabeth M. F. Denton. Published by Walker, Wise & Co., Boston. It purports to be a volume of "Psychometric Researches and Discoveries." It commences with quotations from Newton to Locke and others, and follows these up with tom-fooleries more absurd than any thing ever written or imagined by any body we have ever heard of before. It beats every thing else in the nature of biology, mesmerism, phrenology, steam doctoring, water-cure, and witchcraft. The very queerness of the book will make it be read with profit by cultivated minds. The great point of the volume, as we understand it, is this: it has been said that the human eye, or the retina, retains for a few minutes after death the image of the last person or thing seen when dying. A sweet, a beautiful idea, if true, for often do the dying speak as if they 'saw angels ascending and descending,' and dear kindred and friends who had gone before. So the book argues, that to highly sensitive or impressible persons even the rocks of any locality will reveal the history of all that has passed in their presence, as it were, of which nearly a hundred illustrations are given, purporting to be facts. The lady closes with this practical application: "May we not reasonably hope for less of wrong and more of right, when we shall have learned that all on which our shadows rest become recording angels, faithfully transcribing their deeds, their thoughts, nay, the very motive of our hearts?" But suppose we never "learn" these things, what then?

But let us turn to something better, to books full of practical, tangible truths, all warm with the realities and loves and benevolences of life, such as can be read, with satisfaction and confidence, by happy families around the firesides of dear old Christmas-times; books full of truthful teachings about life and death, and an eternity of blissful realities beyond the grave, and how to attain them with a certainty that "passeth knowledge."

Among such books are some of the issues of the Carter Brothers, publishers and booksellers, 530 Broadway, New-York, to whom the public are so much indebted for the sterling publications which they have been scattering broadcast over the country every year, for almost an age past. Well may that house feel a noble pride when they look over the long list of their issues, not one of them against good morals or against a sound Christian doctrine.

"THE THREE CRIPPLES," pp. 202, 16mo, taken from London life, and is admirably adapted to engage the attention, and instruct the minds, and warm the hearts of children. So is No. 2, entitled "THE LAST SHILLING AND THE OILED FEATHER," by Rev. Philip Bennett Power, A.M. No. 3. "THE TWO BROTHERS AND THE TWO PATHS," by the same, a powerful illustration of what infidels term "Destiny," but which the humble Christian delights in considering the *Providence of God*, and the importance, especially to the young, of seeking the course and guidance of Him who hath said: "It is not in man that walketh to direct his steps." Price fifty cents each. No. 4. "FAITHFUL AND TRUE; OR, THE EVANS FAMILY," 357 pp., 12mo, with nineteen chapters of most interesting incidents of practical every-day life. No. 5. "THE SAFE COMPASS, AND HOW IT POINTS," by Rev. Richard Newton, D.D., 318 pp., 12mo, containing ten practical lectures, admirably adapted to be read of winter evenings around the center-table. Not a child or parent could possibly go to sleep during any one of these stirring readings. Price of last two, ninety cents each. No. 6. "THE JEWISH TABERNACLE AND ITS FURNITURE, IN THEIR TYPICAL TEACHINGS," by Rev. Richard Newton, D.D., 393 pp., 12mo, $1.50. How we long for the return of the good old times when Christian people would feed with delight on books like this, which practically explain the types and the antitypes of the Mosaic dispensation, looking forward to the coming of the Son of Man, his first and his last. No. 7, 12mo, pp. 264, $1, is an "ESSAY ON THE IMPROVEMENT OF TIME," by John Foster, author of that grand essay, on "Decision of Character." This volume is edited by J. E. Ryland, M.A., with a Preface by John Sheppard, and needs no eulogy. Every young man and woman should read it. A bare list of the subjects treated will procure many a purchaser a value of time, capacity of time, swiftness of time, ultimate object of the improvement of time; indolence, intervals, solitary life. We will send this most valuable book, free of charge, to any one who will send us three new subscribers to HALL'S JOURNAL OF HEALTH for 1864.

PRESBYTERIAN BOARD OF EDUCATION, 821 CHESTNUT STREET, PHILADELPHIA.—We wish very much that we could send a catalogue of the books and tracts, with the prices, of this noble Christian institution. What multitudes of luscious feelings would be excited in the young of our land, if half a dozen of these publications could be thrown in upon each family group on Christmas eve, by that famous myth, Chris Kringle, or Santa Claus. Five of the "series for youth," have been sent us for notice, 16mo, averaging 200 pages each. No. 1. "THE THREE HOMES;" 2. "REBELLA; or, The Shining Way;" 3. "Rays of Light"—being instructive tales for youth; 4. "Lessons in Flying," a title which does not convey an adequate idea of the many instructive things found in its pages; 5. "Blind Annie Lorrimer," a sad and beautiful story. These books are from thirty to forty cents each.

Any books we notice, now or hereafter, will be sent to such as choose to take any trouble to obtain subscribers to the Journal of Health for 1864, at the rate of one dollar's worth of books, for three new subscriptions.

The American Tract Society, Boston, and Bible House, New-York, have issued a very interesting and instructive little volume, 16mo, 160 pp., entitled "Plants," illustrating in their structure the wisdom and goodness of God; with numerous engravings. None can read it attentively without benefit to both mind and heart. Also, "Elton Wheatly, the Stammerer; or, Like Other Folks," by Ellen Derry; twenty-five cents. Also, "Happiness," 16mo., pp. 232, fifty cents; being discourses at Geneva, by Count De Gasparin; translated by Mary L. Booth, with an introduction by Rev. E. N. Kirk, D.D., who well remarks: "M. De Gasparin, in this little volume, appears before the American people in a new light; not as a statesman or a political philosopher, but as a moralist and Christian teacher, a disciple of the crucified Redeemer."

Agricultural.—Not only practical and scientific farmers, but the whole country, is greatly indebted to the laborious industry, energy, and judiciousness of the Hon. Isaac Newton, the Commissioner of Agriculture, for his report presented to Congress for 1862, under the act to establish a Department of Agriculture. This is the first report under the new Department, 617 pp., 8vo, with a most copious and satisfactory index of contents. Besides invaluable and suggestive statistical tables, showing the quantity of farm products of different States, there are many articles of practical value, written by eminent men, on the wheat-plant, sorghum culture, cotton, flax, tobacco, fruits, vines, sheep, cattle, eggs, sugar, coal-oil, farm implements, preservation of food and timber. No small part of the value of this report is justly attributed to the persevering industry of James Grinnell, Esq., of Mass., Chief Clerk.

Clinton Hall Literary and Educational Course.—Course of English literature, consisting of readings from Authors, in prose and verse, by Mrs. H. Bronson Williams. This excellent and accomplished lady never appears before a New-York audience without eliciting the heartiest plaudits of her hearers, and none of our readers can fail to derive both instruction and amusement from any one of her "Readings."

The American Sunday-School Union, now venerable for its age and grand in its long years of usefulness, sends out, from 1122 Chestnut street, Philadelphia, and 599 Broadway, New-York, "The Children of Blackberry Hollow," in uniform library binding, of six volumes, in a case, being stories of "Red Shoes," "White Frock," "Tom Lane's Cent," "The Little Brown House," "Little Lights," and "New Bonnet." Also an admirable little volume, "An Appeal to the Young on the Importance of Religion,"

by that eminent writer John Foster, author of "Decision of Character." It is a permanently valuable Christmas present for all young people. Also, "LEONARD, THE LION HEART," "SECOND BOOK OF ONE HUNDRED PICTURES," "THE LITTLE SEA-BIRD," and "THE PEASANT AND HIS GUEST, ILLUSTRATING THE HISTORY OF FOUR BOYS." Any one of these volumes will make the children's eyes fairly dance with delight, if presented around the Christmas-eve fires, with the more solid good of their reading to come after.

POSTURE IN WORSHIP.—An old-school Presbyterian brother and correspondent blazes away most furiously at us for writing Health Tract No. 176, showing that the universal sitting posture during the entire Sabbath service, in cities and large towns, is not only unbecoming, but that, on physiological principles, it unfits the hearer for taking that interest in the services of the sanctuary which all ought to show, simply because it inevitably promotes sleepiness—our correspondent taking his standpoint from a one-acre lot, his own country church; we from New-York, the metropolis of a continent, the head-quarters of all creation. "We speak what we know and testify what we have seen" every day in the churches. The evil has become so general that the General Assembly of our Church has had their attention drawn to the subject, and in obedience thereto, has earnestly urged upon the churches the importaice of breaking up so unseemly a custom. *Our* church on Fifth Avenue numbers nearly eight hundred communicants. We have never been able to number two per cent of the entire congregation who stood during any part of the service, except during the Doxology and the Benediction.

GENERAL NOTICE.—Inducements to subscribe for the JOURNAL OF HEALTH for 1864.

Almost every subscription to this JOURNAL ends with this December number, 1863. We have not a dun to make, as the JOURNAL is not sent unless payment is made in advance. We hope all our present subscribers will find it convenient to renew their subscriptions, and remit the accustomed dollar before the issue of our January number, which will be about the fifteenth of December. And as we have not increased the size or subscription price, although paper and labor have so largely increased in price, we suggest that each subscriber spend an hour or two among his or her friends, in getting new subscriptions for us. We as an editor and physician are often asked and expected to spend hours of valuable time in looking into cases and listening to details and writing letters—for nothing! We make no such request from any reader; but say to them that for three dollars sent for three *new* subscribers, to as many addresses, we will send any book we have published, free of cost; or we will send, post-paid, a dollar's worth of any book or books which we have noticed in this or previous numbers, or we will send, post-paid, the Photographic Magnifier for three new subscribers. This instrument through one glass gives a greatly increased beauty and interest to any photographic picture, and always affords a sweet and quiet delight when looking at the pictures of the loved or lost. When both glasses are used on double pictures—that is, stereoscopic views—the same pleasurable results are experienced. We ourselves never weary in using one of them on the photographs of the members of our family, or the Photographic Albums which contain the cartes de visite of our friends. The Photographic Magnifier is a source of perennial satisfaction and quiet delight to every person who owns one and has the taste or culture to own a photograph album.

Russia and the Russians.—The first article of this number, on the death of Nicholas the First, of Russia, was written by us several years ago for another publication, and is reproduced here as a subject of fresh interest, from the fact that the Russian fleet is in our harbor, and its officers are receiving the cordial hospitalities of the principal cities of the nation. The august Emperor's death was the result of a little cold, which we have private means of knowing was taken by encountering exposures, against the vain remonstrances of a faithful and skillful physician; just as multitudes of our daughters lose their lives by disregarding the counsels which their more experienced mothers give them, in affectionate solicitude, for their highest interest and happiness. "It won't hurt me," says the daughter, as she bounds away to the ball, of a winter's evening, scarcely half-clad. "It won't hurt me," said the "Czar" to his watchful physician, as he went to the "review" of his troops, on that bitter cold day; but let it be understood by all, that potentates and powers; that youth and beauty; that professional eminence and social distinction, are alike subject to the great laws of our being; that the king in his palace, any more than the peasant in his hut, or the slave in his cabin, can not trifle with physiological laws, and that their infraction will always, sooner or later, meet with a due punishment. Many persons, especially clergymen, sometimes encounter exposures, which result in death, under an indefinable impression that their motives or their work will, somehow or other, secure them an impunity against their effects.

We recently attended the religious services of the Greek Church, on the Sabbath-day, on board the Osclaba, and were greatly pleased with the serious and becoming and respectful attention of the four hundred sailors who participated in the services; they stood during the whole time, which was mostly passed in a sweetly plaintive rehearsal of portions of scripture; it was a kind of chanting which fell sweetly on the ear. We shall always remember the scene with great satisfaction and a higher appreciation of Russian civilization and religion. And we are quite sure that our [redacted] daughter [redacted] is more enthusiastic than ourselves at the "civilization" part, having spent eleven hours on board the Alexander Nevsky, the Russian flag ship, the other day and evening, at a grand entertainment given by the officers of the fleet. One of the daily papers, in describing the entertainment, says: "The tenth of November, 1863, will long be remembered by those who participated in the festivities. In homely language, all who were there can not fail to say: We had a splendid time. Nor will it alone be remembered here. The memory of that day will be borne over the sea, taken to Russia, and its anniversary may in years to come be the theme of conversation, on both continents, around the home fireside."

The Russians here are as white as our own sailors and naval officers, of medium height, perhaps rather under that, on an average, with flaxen colored hair, many of them, compact frames, and very courteous; these statements are particularly true of the officers, which is the point of most interest to our young lady readers. The hair is cut short, and the face more or less cleanly shaven.

The grand ball at the Academy of Music was open to any one who was willing to pay twelve dollars. Money could not purchase admission to the festivities on the Russian flag-ship; hence they were characterized by an elevation, a refinement, and a propriety worthy of all praise.

Concentrated Economy.—W. D. Russell, 206 Pearl street, New-York, not only furnishes a stove which will cook a good breakfast for several persons at a cost of two cents, but sells a lamp which will not only furnish abundant light for sewing or study for a whole evening for a penny, but will, at the same time, *without interference*, boil water, make a cup of coffee, chocolate, or tea, and toast bread, *secundem artem.*

Farmers.—The most extraordinary case of success in an enterprise that is unquestionably of sterling and permanent and general utility, is found in the energy and business tact of Orange Judd, the editor and proprietor of the *American Agriculturist*, New-York, $1 a year. When he took hold of it, it had but a few hundred subscribers, and was dying daily; now it has a circulation of fifty thousand greater than any similar publication in the world. It is sent monthly to ninety thousand actual subscribers. Whoever wants it for nothing, and has never taken it, let him send three dollars for three new subscribers to Hall's Journal of Health.

To Every Lady of taste and culture; to every housekeeper; to every mother, *Godey's Lady's Book*, $3 a year, Philadelphia, is of sterling value. The industry, ability, and judgment with which it is edited, is worthy of all praise, and has made it the most popular family monthly in the nation. January, 1864, begins the sixty-eighth volume.

Photographic.—We never fail to be pervaded with a deep and quiet and intense satisfaction when we look at the photographs of our dear little ones, or of the loved and absent, through the *Photographic Magnifier* and Pocket Stereoscope sold by P. C. Godfrey, 831 Broadway, New-York, at $1, $1.50, and $3. Sent by mail free at same prices.

Rewards.—The two bound volumes of Hall's Journal of Health for 1862 and 1863, containing, among other reading pertaining to health and disease, all the "Health Tracts" published to this date, one hundred and eighty in number, can be had for $1.25 each: they will be given to any one who will obtain seven new subscribers for our Journal for 1864.

Subscribers who have failed to receive any number of the Journal for 1863, will be supplied with the same free of charge on application by letter, or at the office of publication, 831 Broadway, New-York. Any past number of the Journal, from No. 1, Vol. I. to date, will be supplied for ten cents.

The two volumes for 1862 and 1863, containing one hundred and eighty "Health Tracts," bound in muslin, will be delivered at the office of publication for two dollars, or sent by express at purchaser's charge for the same amount.

HELP FOR WOMEN.—Messrs. Walker, Wise & Co., 285 Washington street, Boston, have issued a timely volume for $1.25, on "THE EMPLOYMENTS OF WOMEN," being a Cyclopædia of Women's Work, by Virginia Penny. At no time of our country's history have so many women been thrown on their own exertions. The book contains over five hundred articles descriptive of occupations available to women, the effect of each on health, rate of wages, time necessary for women to learn them. It is an invaluable work, a work of humanity, and precisely adapted to the emergencies of the times. We will send it post-paid to any one who will send us three new subscribers to HALL'S JOURNAL OF HEALTH for 1864.

The bound volume for 1863 will be given for the loose numbers (if in good condition) and twenty-five cents for binding. We shall probably issue the subsequent numbers in white paper binding, each number containing about twenty-four pages of reading matter; the extra pages will be devoted to advertisements.

Seven copies of the JOURNAL OF HEALTH for 1864 will be sent for five dollars sixteen copies for ten dollars. Ladies who desire to consult the Editor professionally, are requested to do so by a prearrangement of the hour.

The Presbyterian Historical Almanac for 1863 should be in the library of every clergyman in the whole Presbyterian family; it embodies a vast amount of important statistical and historical matter, beside the biographies and portraits of ministers who lived and labored and loved and died in their Master's service. This, the fifth volume, contains nineteen ministers' portraits, engraved upon steel by Sartain and Whitchurch; also eight engravings of churches, colleges, and seminaries; 495 pages 8vo, $2. Either of the first four volumes will be furnished for $1.50, by addressing its indefatigable and self-sacrificing compiler, Joseph M. Wilson, No. 111 South Tenth street, Philadelphia.

The following was taken verbatim from a tomb-stone at Williamsport, Pa., last summer, by a son of Rev. Dr. R——s of New-York:

Sacred to the<br>

memory of<br>

HENRY HARRIS,<br>

Born June 27, 1721.<br>

of Henry Harris and Jane<br>

His wife, died on the 4th of<br>

May, 1737, by the kick of a<br>

Colt in his bowels, peaceable<br>

And quiet, a friend to his<br>

Father and Mother, and respected<br>

By all who knew him.<br>

And went to that world<br>

where horses can't kick, and<br>

where sorrow and weeping<br>

is no more.

